# Some American Methods
**A Review of the American Hospital.* By Edward F. Stevens. (New York: Architectural Record Publishing Company. 1917. Price $5.00.)


**Published:** 1918-11-30

**Authors:** 


### HOSPITAL CONSTRUCTION AND PLANS.

### Some American Methods.*

About seventy hospitals are illustrated in this work by Mr. Stevens. For that reason, if for no other, the book would be of value, for a synoptic collection of examples of buildings of a special type is always of interest and use to those who are engaged in the construction or the study of similar erections. The illustrations are chiefly plans, and as such would be of much greater value if uniform care had been taken to see that every plan had an intelligible scale attached to it. Some of these illustrations have no scale at all—*e.g.* Mr. Stevens's own plans of the East New York Hospital; others have a scale printed so small (owing to reduction) that no eye, even with glasses, can read it; and one plan at least bears the announcement that it was, presumably *before reduction*, drawn to the scale of some fraction of an inch to the foot. As the information is of no use to the reader it scarcely matters that the fraction is indecipherable.

One general remark in regard to all the American plans in this book will spring to the lips of every English architect who sets eyes upon them. He will observe that the problem set before the American architect is immeasurably simpler than that with which we in this country contend, and he will be tempted to say, perhaps "in his haste," that if access of light and transmission of fresh air are *not* to be taken into account the main crux of hospital design no longer exists. Indeed, it is in the violation of all our canons of air and light that these American types differ chiefly from our English designs. Akin to this observation and perhaps connected with it is the fact that the Americans put into their general hospitals many more single-patient rooms than prevail in our institutions, and many more small wards of three or four beds. We mention this as akin to the light and ventilation problem, because the first thing that occurs to an architect in England when he finds that he has to deal with the small-ward problem is that he is almost bound to violate his conscience by abandoning or mitigating his principle of cross-ventilation. It is possibly in despair at the difficulty of ventilating these small chambers by natural means that the American frankly gives up the attempt; but it is strange indeed that he should go the length of giving approach to these rows of single-bed rooms by long central corridors which often have no windows at all, or at best a window at each end of 60 or 70 yards of passage. The reader will, moreover, look almost in vain among these designs for anything approaching the sanitary block (with cross-current cut-off) which is one of our cardinal virtues. Perhaps we are unduly devoted to this European fancy. Mr. Stevens tells us, indeed, that some of our leading authorities are losing faith (or superstition) on this point, and he alludes to the feature in gentle derision as "the old English toilet tower." He admits that it had excellent reasons for its existence, though he considers it had an undue tendency to darken the wards. One example of it appears in the book, that of the San Francisco Hospital, and the writer notes it as a rather obsolete peculiarity.

Presumably the isolation of sanitary fittings, the daylight illumination of corridors, and the general circulation of natural atmosphere are so far left behind by the scientific advances of medical hygiene in the States that the present position as regards such matters is hardly worth explaining; otherwise we should have wished to get from Mr. Stevens some fuller statement of the arguments against the use of fresh air and natural light. It is true he explains briefly that the rugged climate of Northern America forbids the complete disconnection of block from block which is found in European hospitals, but this is part of an argument for the use of covered corridors, and does not really affect the question of internal fresh air in the blocks themselves. In a few pages Mr. Stevens deals specifically with the ventilation of hospitals, but the subject is all too briefly dismissed, for it seems obvious that unless a Plenum system is generally adopted—and the writer makes it clear that it is not—many of the hospitals here illustrated must inevitably suffer deleteriously from the undue circulation of exhausted and infected air. It is made clear, of course, that most of these buildings are fitted with elaborate systems of flue-extract, and one gathers that in most cases reliance is placed on powerful mechanical exhaust. But when all this is explained or implied the English architect still shudders at the sight (on a plan) of those water-closets tucked in among the bedrooms, of the private wards that have such conveniences directly attached to them, and finally at such amazing phenomena as the group of four w.c.s and a bathroom (fig. 51), which have not got a window among them all!

It is perfectly true that there is room for two schools of thought on this subject, and it is easily arguable that if you are going to trust the plumber at all you had better trust him completely and take all your sanitary fittings into confidence. But there is more in this than a mere question of traps, and we are inclined to believe that the tendency in American private life to the overheating of houses is paralleled in the hospitals by an attitude of indifference to the inexpensive blessings of God-made air and Heaven-sent light. Against these suggestions, which are only the mere contrasting of opinions American and English, one must acknowledge that the open-air treatment is very liberally catered for by our American friends. The open-air ward, airing-balcony, and solarium are very important features of many, nay most, of these designs. The Transatlantic idea would seem to be "When you are out of doors be out of doors, but when you are indoors don't unduly worry about fresh air. What is a house for except to keep you warm as well as dry?"

"The day of the amphitheatre as an operating unit for teaching seems," says Mr. Stevens, "to have gone." It went in England a long time ago, and its disappearance was a great boon. The illustration, however, of the Massachusetts Hospital shows one of the old type, and presumably of the old size, though the absence of a scale makes it difficult to test this, and the plan of Mr. Stevens's Grace Hospital, Detroit (again no scale), seems to indicate a large room with the once familiar tiers of students' platforms. On white walls for operating-rooms Mr. Stevens is very severe. In America the objection to white is apparently very acutely felt on account of eye-strain to the surgeons, who, not content with prescribing a very dark green for the floors and dados of their operating-rooms, also "use dark gowns for themselves and attendants."

Mr. Stevens's design for a nurses' residence at Bridgeport is a fine and simple building, but in it again one

* *A Review of the American Hospital.* By Edward F. Stevens. (New York: Architectural Record Publishing Company. 1917. Price $5.00.)

**American Methods**—(*continued*).

sees how easy life is made for the American architect by the discovery that windows are not necessary for sanitary rooms. It is an L-shaped building, and consequently has a "re-entering angle," that bugbear which it is so difficult for the architect to cope with. The American architect has no trouble at all. Here, he says, is a place in my plan with no possibility of windows. What shall I put there? Why, *baths*, of course; so in go four bathrooms, two and two, with a lobby between them. One bathroom has a light, the other three and the lobby none! The Nurses' Home at St. Luke's Hospital, Jacksonville, has halfway along its windowless corridor (say 140 feet long, in the absence of a scale) a "toilet room" about 20 feet by 15 feet. This is an apartment divided up into a washing space with five basins, three bathrooms, and as many w.c.s; over the basins are two windows, and there is one window in one of the bathrooms, but everything else is windowless!

We *know* this is all as it should be, for our American friends and rivals are well in the front for sanitary knowledge; but it sets us wondering whether we are merely old-fashioned in our British prejudices, or whether, in refusing to simplify half our difficulties by casting ourselves on to the mercy of artificial ventilation and artificial light, we are not after all doing right.

This article must contain at least a mention of the pages at the end of the volume, which give plans and other details of some military hospital designs for the U.S. Army. These are very interesting, and exhibit the marvellous completeness and—shall we say?—opulence of the provision which the Americans made for their medical and surgical needs in the war.

In the alleged absence of any other history of American hospital construction we can welcome this little book as distinctly a useful record, but perhaps even Mr. Stevens would hardly be satisfied to have it regarded seriously as a standard work on the subject. It contains eighteen chapters, to be sure, but as the total of pages only runs to 274, and as the paper is largely covered with illustrations (many pages containing no text at all), these chapters do not average ten pages each. We should scarcely mention this point in reference to a book which the author may not have intended to be more than a collection of reprinted plans with cunning commentary, but that the publisher's circular which heralded its appearance takes the whole affair in a much more serious light. Intending purchasers reading of the "vast fund of correlated facts," of the "exhaustive research," of the aid given by "prominent specialists," and of the probability that this work will "remain a standard authority" may feel some disappointment over their bargain, especially when they are assured by the publisher that the author (whose biography and list of works are appended) "is known throughout Europe and America as the leading architectural authority on hospital construction and equipment." Mr. Stevens has had various partners, and has often been joined with others as consulting architect; it is not therefore easy to criticise with praise or blame his work as a designer of elevations. The published designs to which his name is attached are strangely variant in standard of beauty, some are distinctly commonplace, others are quite good and nearly worthy of the man "whose specialised genius is represented by some of the most perfected and noblest edifices extant among modern hospitals."

